# The CogLearn Toolkit for Unity: Validating a virtual reality paradigm for human avoidance learning

**DOI:** 10.3758/s13428-025-02630-5

**Published:** 2025-04-29

**Authors:** Marina Rodriguez Lopez, Huaiyu Liu, Federico Mancinelli, Jack Brookes, Dominik R. Bach

**Affiliations:** 1 UCL Queen Square Institute of Neurology, Wellcome Centre for Human Neuroscience, University College London, London, UK; 2 Centre for Artificial Intelligence and Neuroscience, Transdisciplinary Research Area Life and Health, University of Bonn, Bonn, Germany

**Keywords:** Human avoidance learning, Virtual reality paradigm, Naturalistic measure, Toolkit

## Abstract

Avoidance learning encompasses the acquisition of behaviours that enable individuals to evade or withdraw from potentially harmful stimuli, prior to their occurrence. Maladaptive avoidance is a crucial feature of anxiety and trauma-related disorders. In biological and clinical settings, avoidance behaviours usually involve uninstructed, idiosyncratic and complex motor actions. However, there is a lack of laboratory paradigms that allow investigating how such actions are acquired. To fill this gap, we developed a wireless virtual reality platform to investigate avoidance learning in naturalistic settings, with an uncomfortable sound as unconditioned stimulus (US), a physically plausible avoidance action, and allowing for unconstrained movements. This platform, the CogLearn Toolkit for Unity, is publicly available and allows conducting various types of learning experiments with simple text files as input. We validated this platform in an exploration-confirmation approach with five independent experiments. Overall, participants showed successful acquisition of avoidance behaviour in all experiments. In three exploration experiments, we refined the paradigm and identified mean distance from US location during conditioned stimulus (CS) presentation (before US occurs) as a sensitive measure of avoidance. Two confirmation experiments revealed stronger avoidance for CS+ than CS- during avoidance learning, whether or not this phase was preceded by Pavlovian acquisition. Furthermore, we demonstrated reduced avoidance during extinction with instruction to approach CS, but persistent residual avoidance during this phase. We found evidence of reinstatement in one of two confirmation experiments. Overall, our study provides robust evidence supporting the efficacy of our paradigm in studying avoidance learning in conditions of high ecological relevance.

**Supplementary information:**

The online version contains supplementary material available at 10.3758/s13428-025-02630-5.

## Introduction

Avoidance learning is a type of instrumental conditioning in which a certain object or spatial location (conditioned stimulus, CS) is coupled with a noxious event (unconditioned stimulus, US) such that the agent can avoid US exposure by performing (or withholding) certain actions. Avoidance learning is paramount for any species that inhabits dynamically changing environments and must protect themselves from harm such as predators (Colwill, 2022). However, maladaptive avoidance, e.g. avoiding non-harmful objects or places, could be detrimental. Indeed, dysfunctional avoidance is observed in various psychiatric conditions, including post-traumatic stress disorder (Yehuda et al., 2015), anxiety disorders (Craske et al., 2017), and obsessive-compulsive disorder (Stein et al., 2019). As such, avoidance learning is often investigated in a pre-clinical context, and in paradigms designed to capture specific aspects of clinical conditions (for a review, see Krypotos, 2015). Specifically, maladaptive and enduring persistence of avoidance can be instigated directly by the process of avoidance learning (Moutoussis et al., 2008). This is because performing the avoidance action not only prevents US exposure, but often also blocks any information on whether the US actually occurs, which precludes further learning. Thus, if avoidance erroneously takes effect in situations that are actually safe (e.g. because of improper CS generalisation), or if avoidance ceases to be adaptive because CS-US contingencies have changed, then the agent would be unable to adapt their learned actions. In clinical conditions, this could mean that agents abstain from potentially rewarding situations and/or are confined to performing costly actions with no objective benefit (e.g. Moutoussis et al., 2008). Hence, the computational, algorithmic and neural underpinnings of avoidance learning are of obvious bearing on issues of clinical relevance.

A variety of laboratory paradigms in humans have been developed to investigate such issues, including the role of avoidant habits (De Wit et al., 2018; Flores et al., 2018; Godier et al., 2016; Patterson et al., 2019; Roberts et al., 2022) and of avoidance generalisation (Glogan et al., 2022; Lemmens et al., 2021; Norbury et al., 2018; San Martín et al., 2020; Wong & Pittig, 2022). While thought-provoking and informative, currently existing experimental procedures could be improved in several directions (Krypotos et al., 2018; Pittig et al., 2018). (1) The situations faced by participants sometimes lack in ecological validity; both in terms of the eliciting situation and in terms of the required avoidance action. For such simplified paradigms, clinical relevance remains to be established. (2) Avoidance is often implemented by recurring categorical (mostly unary or binary) measures, which precludes assessing the clinically relevant spectrum between a weak and a strong avoidance response. (3) Unlike in biological or clinical situations, instructions often play a major role in shaping avoidance learning, and in particular in finding the required actions. Presumably, these shortcomings come about because the vast majority of tasks (with due exceptions, e.g. Reichenberger et al., 2017) take place on a computer screen. This often (though not always) implies that the required actions are non-natural, often discrete (e.g. key presses), and have no intrinsic relation to the stimulus that the agent seeks to avoid, such that they need to be instructed rather than found by exploration. Finally, this precludes body movement measurements, which might be pertinent to naturally and clinically occurring avoidance actions and would arguably add to the granularity of avoidance measures.

In a bid to overcome these issues, the present work introduces a novel task in immersive (wireless) virtual reality in order to study human avoidance learning. The task is presented and validated here in its basic form, but the paradigm can be modularly extended to study complex learning phenomena or computational models. It simply requires participants to learn to avoid a noxious sound by moving in a virtual room. Participants come up by themselves with the timing and execution of their avoidance action and are uninformed about experimental contingencies. We then measure where, when, and which, avoidance responses occur. In the remainder of this manuscript, we introduce the software tool used to generate experimental paradigms, evaluate a range of outcome measures, establish their retrodictive validity (Bach et al., 2020) in independent confirmation experiments, and discuss future applications.

## Methods

### The CogLearn Toolkit for Unity

Our goal was to create a versatile avoidance learning platform within the Unity VR game engine with the following conceptual features: (1) a primary reinforcer as US that can be implemented in wireless VR; (2) a conditioned (avoidance) response that is physically related to the specific US and can be found by free exploration; (3) full flexibility to present single or multiple objects as CS; (4) a possibility to implement context conditioning (not used or evaluated in the current work); (5) a possibility to implement an incidental task during which avoidance can be quantified; (6) definition of experiments by means of simple text files without the need to train in the Unity software.

**Fig. 1:**
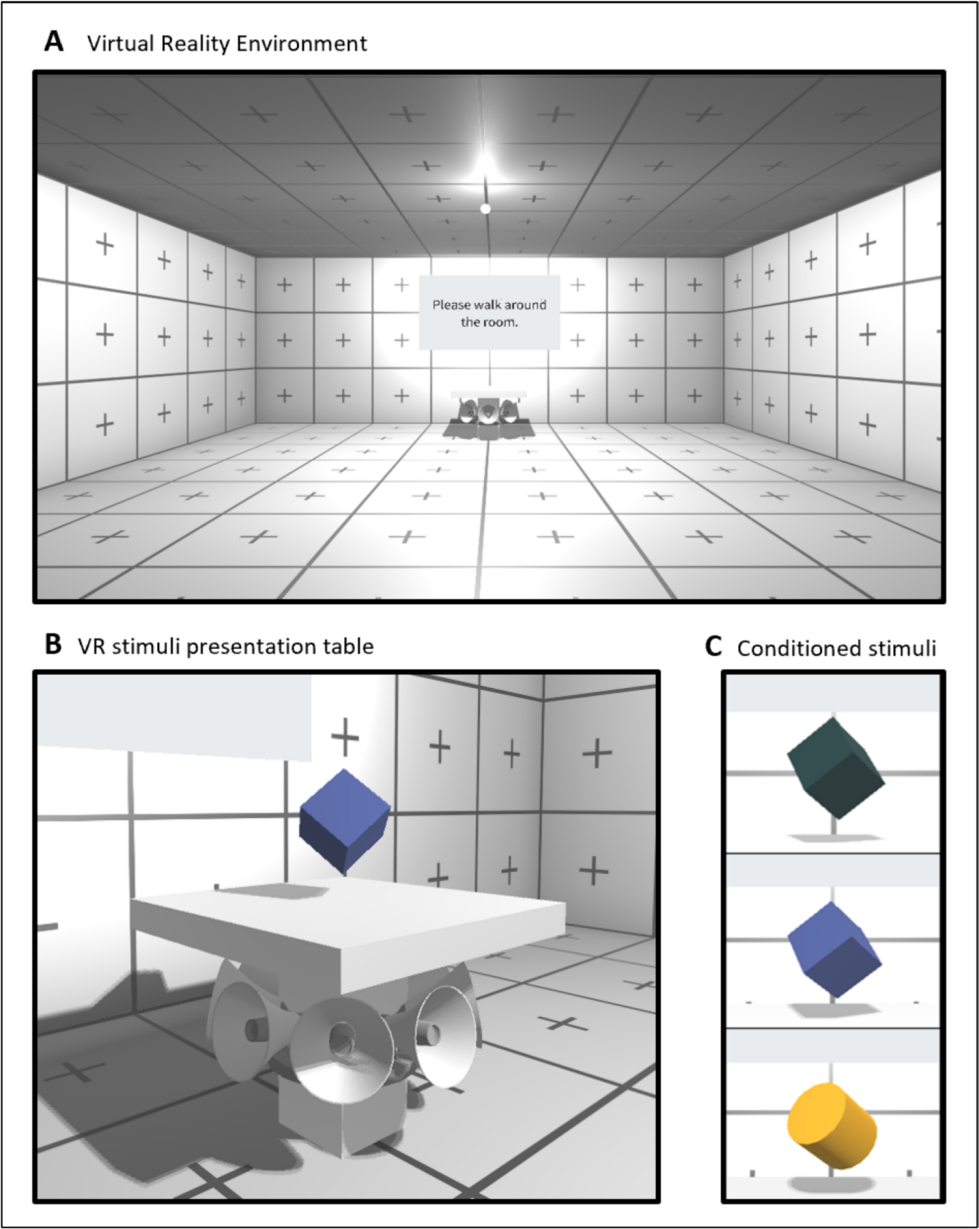
**A** Virtual reality environment. The screen, pedestal and small lightbulb are the only objects in the virtual room*.* **B** The pedestal on which CS is presented. In experiments 2–5, a set of speakers underneath the pedestal vibrate when US is presented. **C** Conditioned stimuli. In experiments 1–4, CS were the *green* and *blue cubes*, while in experiment 5, CS were *blue cube* and *yellow cylinder*

Thus, we created the CogLearn Toolkit for Unity, which includes the following features:

1. *Virtual room*. Throughout the experiment, participants are located within a bare, tiled, square-shaped room (dimensions: 8 m x 8 m x 3 m; see Fig. 1A). Tile pattern was visually designed to provide clear orientation cues and to minimise cybersickness compared to plain walls in a series of technical tests. Room colour can be set for each trial, such as to implement simple form of contexts, and can be changed within the trial for higher-order learning paradigms. At the far end of the room, a screen is mounted where short user-defined prompts can be presented.
2. *US*. The US is a loud monaural sine sound (1760 Hz, 80 dB at zero distance from source, adjustable linear decay from source), which is presented for an adjustable duration. We chose the maximum loudness to be clearly aversive to most participants. The monaural presentation and (non-natural) linear decay were chosen for technical simplicity and because they provide a clearly defined distance from which the US cannot be aversive, whereas a physically realistic inverse-square decay would render this distance dependent on a participant’s hearing abilities. For this type of sound, it is objectively impossible for a stationary person to locate its source. We speculate that this might be the reason why some participants did not exhibit any avoidance response in experiment 1 reported here. To overcome this limitation and signal the sound source visually, from experiment 2 onwards, we added a set of loudspeakers in the sound source location (under the CS pedestal), which vibrated when the US occurred. To prevent participants from garnering visual information about the occurrence of the US, this vibration feature could be turned off.
3. *CS*. In an adjustable location within the virtual room, one or two pedestals provide a platform to present any CS that are defined within Unity (only one pedestal is used in the experiments presented here). The toolkit includes a range of simple geometric objects as CS; new CS can be added in Unity. Multiple CS can be presented on the same pedestal by bundling them into a single Unity object.
4. *Search task*. As an incidental task conceptually based on Binder & Spoormaker's (2020) fishing task, we implemented a search task in which coins appear in succession for 1 s each. They could appear anywhere at random within an elliptical area of 2 m in width and 2 m in depth, 1 m from the floor. The centre of the area is between the subject starting point and the table (1 m in front of the player's starting point). Participants would have to touch the coins with the hand controllers to collect them.
5. *Synchronisation with peripheral equipment*. In order to synchronise with equipment such as psychophysiological recordings, Unity sends a set of transistor-transistor logic (TTL) markers at each event.
6. *Stimulus control*. To define the various adjustable features described above, users can define fixed features in a json file, and trial-specific features in a CSV file, both of which are read and compiled by the CogLearn project at runtime. Multiple definition files (e.g. corresponding to different trial orders) can be provided simultaneously, and the user will then be prompted to select one when starting the experiment.
7. *Data logging*. For trial management and data collection, the toolkit uses the Unity Experiment Framework (UXF, Brookes et al., 2020). Trial-wise data are saved in CSV format with one row per trial. Movement data from the head-mounted display (HMD) tracking system, sampled at the rate that the Unity simulation runs, which is currently tied to the render rate (80–120 Hz, depending on HMD), are reported in Unity’s left-handed coordinate system and saved as one CSV file per tracker per trial. A number of functions to read and manipulate this type of data are provided with our R packages CogLearn (https://github.com/bachlab/CogLearn) and vrthreat (http://github.com/bachlab/vrthreat) (Brookes et al., 2023; Sporrer et al., 2023).

This toolkit is available on OSF (https://osf.io/8u9ms/), both as build (not requiring Unity) and as Unity project. With the build, the user can change parametric features of the task, such as CS type, US parameters, stimulus timings, pedestal number and positions, room colours, prompt texts, task breaks, and features of the search task (via json and CSV files). This allows building many types of associative learning experiments, such as delay, trace and context conditioning with various timing and reinforcement settings, return of fear tasks, latent inhibition, second-order conditioning, or Pavlovian-to-instrumental transfer tasks. By simply adding new CS objects in Unity, the user can implement further associative learning experiments requiring compound CS or generalisation stimuli, such as forward and backward blocking, overshadowing, summation tests, patterning tasks, and generalisation experiments. The Unity project also allows users to change the conceptual layout of the task, and can thus serve as starting point for more varied learning experiments.

### Participants

For the reported five validation experiments, non-overlapping samples of healthy individuals were recruited from the general population through university-wide recruitment platforms (see Table 1 for demographic details and Table 2 for a summary of questionnaire scores) and received a fixed monetary compensation. Volunteers were considered eligible to take part in the study if they met the following criteria: over 18 years old; fluent in English; no history of neurological, psychological or medical conditions; no movement impairments; normal hearing, and normal or corrected-to-normal vision. Across all experiments, seven participants (< 5%) did not complete the experiment per protocol due to technical failures and were therefore excluded for analysis. For all experiments, we conducted sensitivity power analyses for one-sided paired *t* tests (CS+ > CS-) using G*Power 3.1.9.7 (Faul et al., 2007), assuming 80% power and an alpha level of 0.05. These analyses showed that our samples were powered to detect effects with Cohen’s *d* values of 0.51, 0.50, 0.44, 0.45, and 0.45 for the five experiments, respectively. All participants gave written informed consent before the experiment, in accordance with the Declaration of Helsinki. The experiment, including the form of establishing consent, was approved by UCL Research Ethics Committee (6649/005).

**Table 1:** Demographic information for participants that completed the experiments per protocol

Experiment	Sample size	Mean age ± SD (range)	Female/male
1	23	32.4 ± 9.19 (23–58)	13/10
2	24	29.3 ± 10.28 (18–45)	17/7
3	32	23.9 ± 6.36 (19–46)	28/4
4	30	22.5 ± 4.60 (18–40)	23/7
5	31	23.4 ± 6.26 (18–42)	29/2

**Table 2:** Summary statistics (mean ± SD) of demographics and questionnaire scores from all experiments

Questionnaire	Experiment 1	Experiment 2	Experiment 3	Experiment 4	Experiment 5
BMI	23.6 (± 3.44)	22.8 (± 4.71)	21.6 (± 4.50)	20.9 (± 3.02)	21.4 (± 2.37)
BSSS	23.7 (± 5.99)	24.1 (± 5.61)	22.5 (± 5.66)	23.2 (± 5.17)	21.3 (± 6.60)
DPSS	27.5 (± 6.27)	26.8 (± 6.12)	30.1 (± 7.19)	28.0 (± 6.77)	29.7 (± 8.58)
FSS	45.3 (± 20.9)	47.0 (± 28.3)	59.5 (± 35.0)	47.8 (± 23.3)	69.3 (± 22.5)
STICSA-T	27.7 (± 6.53)	28.8 (± 8.07)	33.6 (± 8.06)	30.0 (± 8.86)	32.4 (± 9.26)
STICSA-S	27.1 (± 5.73)	26.8 (± 8.28)	28.2 (± 7.89)	25.4 (± 4.23)	25.8 (± 5.59)
SSQ	16.2 (± 22.2)	19.0 (± 24.7)	27.0 (± 26.7)	24.4 (± 21.9)	21.2 (± 23.2)
Videogames (h/week)	2.26 (± 4.52)	2.55 (± 6.37)	2.31 (± 4.73)	2.64 (± 4.07)	4.07 (± 9.66)

*Note*: *BMI* body-mass-index, calculated from the weight and height responses given by participants days in advance. *BSSS* Brief Sensation Seeking Scale, *DPSS* Disgust Propensity and Sensitivity Scale, *FSS* Fear Survey Schedule. *STICSTA-T/S* State-Trait Inventory for Cognitive and Somatic Anxiety – Trait/State, *SSQ* Simulator Sickness Questionnaire. See main text for details

### Settings and equipment

#### Virtual reality presentation

The VR paradigm was presented on an HTC Vive Pro Eye HMD headset with integrated headphones, using a wireless adapter and run on a PC with an Intel i7 9700K CPU and Nvidia RTX 2080Ti GPU using SteamVR version 1.26.7. Participants held VIVE hand controllers. The experiment was built using the Unity Engine version 2020.3.15f2 (Unity Technologies) under Windows 10 Enterprise (version 22H2).

#### Behavioural data recording

Participants were instructed that they were free to move around and explore the virtual room. The VR equipment allowed unrestricted body and head rotations, including but not limited to arm stretching and motion, jumping, and running. Hand and finger movements were restricted due to the hand controllers being held. Headset and hand controller positions were tracked throughout the experiment.

#### Questionnaire data

We implemented questionnaires using the REDCap electronic data capture tools hosted at University College London (Harris et al., 2009). A few days prior to the experiment, participants provided demographic information, including sex and gender, age, body weight and height. Then, they were asked to complete a set of questionnaires assessing trait anxiety (State-Trait Inventory for Cognitive and Somatic Anxiety, STICSA-T) (Ree et al., 2008), sensation seeking (Brief Sensation Seeking Scale, BSSS) (Hoyle et al., 2002), disgust propensity and sensitivity (Disgust Propensity and Sensitivity Scale, DPSS-12) (Fergus & Valentiner, 2009) and fearfulness (Fear Survey Schedule-III, FSS) (Wolpe & Lang, 1964). We used one question from the Video Game Usage Questionnaire (Tolchinsky, 2013) to assess the participants’ video game habits, specifically asking to indicate the number of hours spent playing videogames per week. Upon arrival, and immediately before the experimental session, participants completed a questionnaire on their state anxiety (STICSA-S: Grös et al., 2007; Ree et al., 2008) and physical state (e.g. hunger, thirst, tiredness, etc.). After the experimental session, participants completed the 16-item cybersickness inventory (Simulator Sickness Questionnaire, SSQ) (Kennedy et al., 1993). Table 2 summarises the sample characteristics for all experiments.

### Stimuli and procedure

#### Virtual reality environment

All experiments used the same virtual reality room. In experiment 1, the only objects in this room were a white pedestal and a screen on which short prompts were presented (Fig. 1A). In experiments 2–5, a set of loudspeakers underneath the pedestal (Fig. 1B) would vibrate slightly when the US was presented to create the impression that the sound was coming from them.

#### Stimuli and timing

For experiments 1–4, CS were differently coloured cubes (blue, RGB: 46, 61, 124; dark green, RGB: 5, 31, 32; size: 0.2 m x 0.2 m x 0.2 m), presented on top of the pedestal. CS-colour relation was counterbalanced across participants. To facilitate CS differentiation and reduce potential generalisation in experiment 5, we substituted the dark green cube with a yellow cylinder (RGB: 222, 151, 11; size: 0.24 m x 0.1 m x 0.24 m) (Fig. 1C). CS were presented for 9 s.

US was a 1-s loud monaural sine sound (1760 Hz) with an intensity of 80 dB at zero distance from source, with a 25% linear decay per meter (becoming inaudible at 4-m distance from the source). US co-terminated with the CS.

The inter-trial interval (ITI) was randomly drawn from a uniform distribution between 9 and 15 s. Thus, mean ITI duration was 12 s.

#### Task sequence

**Fig. 2:**
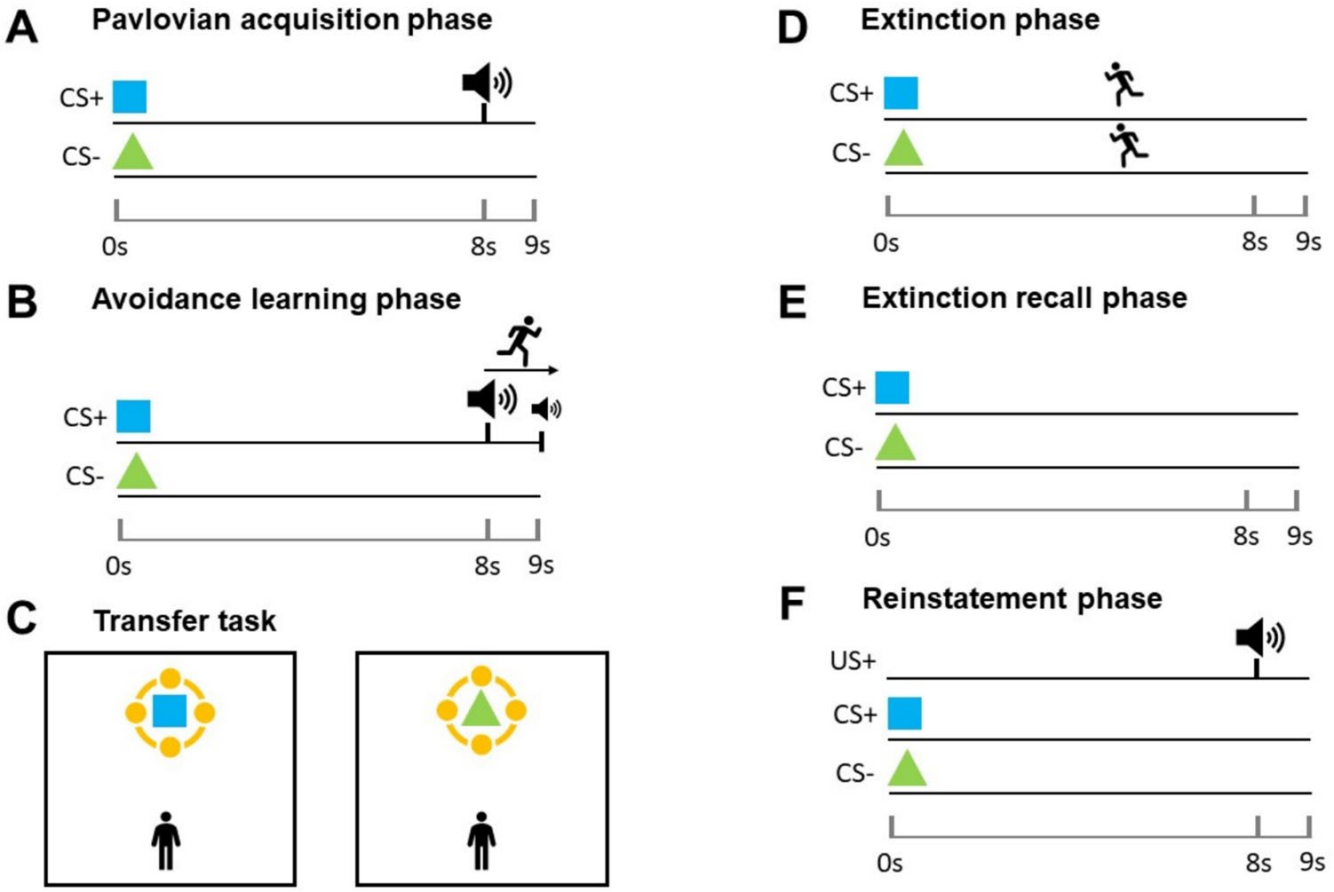
Task sequence diagram. See section task sequence for detailed descriptions of each phase

All five experiments followed a similar design, consisting of different combinations of the following experimental phases: practice phase, Pavlovian acquisition phase (Fig. 2A), avoidance learning phase (Fig. 2B), transfer task (Fig. 2C), extinction phase with instruction to approach (Fig. 2D), extinction recall phase (Fig. 2E), reinstatement phase (Fig. 2F). During breaks between the phases, a prompt appeared on the screen (“Now take a short break”), and the instructions for the next phase were read out to the participant. The next phase would commence after participants confirmed they understood the instructions. Table 3 summarises the trial structure for each experiment.

1. Practice phase: During a 40-s familiarisation period, participants could walk around freely, without any CS being presented.
2. Pavlovian acquisition phase: This phase consisted of 16 trials: 8 CS+ and 8 CS-. Each CS was presented on top of the pedestal for 9 s. In 75% of CS+ trials, a US was delivered 8 s after CS onset, and co-terminated with CS presentation. During the entire phase, participants were sitting in a chair 2 m away from the pedestal, looking towards the CS, and were asked not to move.
3. Avoidance learning phase: This phase had the same procedure and reinforcement schedule as the Pavlovian acquisition phase, but participants were standing and allowed to walk around the room. Trials would start as soon as participants positioned themselves on a starting point, located at 1 m distance from the CS and indicated with a green light and floor mark. Thus, participants were compelled to walk away from the CS if they wanted to avoid the US sound.
4. Transfer task: Participants completed the aforementioned search task while CS were presented for 21 s without reinforcement. Each transfer task consisted of four trials (2 CS+, 2 CS-) in random order. Trials would start as soon as participants positioned themselves on a starting point located 2.5 m away from the CS.
5. Extinction phase with instruction to approach: This phase had the same trial structure as the Pavlovian acquisition and avoidance learning phases (eight trials per condition) but without any US. Trials started as soon as participants positioned themselves on a starting point located 4 m away from the CS. A screen prompt behind the pedestal tasked participants to approach the CS and try to stand as close as possible.
6. Extinction recall phase. This phase consisted of four trials (two per condition), without any US or instruction, and with the starting point located 1 m away from the CS as in the avoidance learning phase.
7. Reinstatement phase: One presentation of the US without CS was followed by trials without US (experiment 4: 16 trials, 8 CS+, 8 CS-; experiment 5: six trials, 3 CS+, 3 CS-). The starting point was located 1 m away from the CS.

**Table 3:** Summary of the experiment structure and task sequence of all five experiments. The trial number refers to the total number of trials, half of which involved CS+ and CS-, respectively

Experiment	Pavlovian acquisition	Avoidance learning	Transfer task 1	Instructed extinction	Extinction recall	Transfer task 2	Reinstatement
1	16 trials	16 trials	4 trials	16 trials		4 trials	
2	16 trials	16 trials	4 trials	16 trials		4 trials	
3		16 trials	4 trials	16 trials		4 trials	
4	16 trials	16 trials	4 trials	16 trials		4 trials	1 US+ 16 trials
5		16 trials	4 trials	16 trials	4 trials	4 trials	1 US+ 6 trials

In all phases except the Pavlovian acquisition phase, participants were free to move around the room.

### Data analysis

All data analysis was conducted using the statistical software R 4.1.0 (R Core Team, 2022). Anonymised trial-level summary statistics and R scripts are publicly available on OSF (https://osf.io/yxvfz/). Full movement data are available upon request under a data sharing agreement in line with local data protection regulations.

### Data pre-processing

Behavioural data were pre-processed using the R packages CogLearn (https://github.com/bachlab/CogLearn) and vrthreat (https://github.com/bachlab/vrthreat). For the continuous head tracker data, we extracted seven trial-level summary statistics over the interval from CS onset to US onset: mean/maximum/minimum distance to CS location; overall distance travelled; maximum speed; head direction relative to CS; reaction time for the first move away from CS, defined as the first time the head moved at least 0.7 m away from its position at CS onset.

### Statistical analyses

Experiments 1–3 served to develop and validate the behavioural task, and to identify the dependent variables that are most indicative of avoidance learning and extinction. The large number of alternative dependent variables that could have been explored poses a considerable multiple comparison problem, such that we focus on effect sizes and report statistical tests for illustration only. Confirmation experiments 4–5 served to test out-of-sample generalisation of results garnered in experiments 2–3.

To assess the CS+/CS- difference within each phase, our primary criterion for the selection of dependent variables, we report effects sizes as Cohen's *d* and Hedge’s *g*. Next, we ran one linear mixed-effects model (LMM) per dependent variable per phase of the experiment. This model included the main effects of the CS condition (two levels: CS+/CS-), trial number, and their interaction, as well as a random participant intercept. We note that models with more complex random-effects structures failed to converge for all available optimisers. As robustness analysis, we ran repeated-measures ANOVAs. Results are available on OSF and largely consistent with LMM results. To compare across phases (acquisition/extinction and extinction/reinstatement), we conducted linear mixed-effects models across phases. Table 4 lists the model syntax in R. We fitted all LMMs and obtained relevant *p* values using the R package LmerTest 3.1.3 (Kuznetsova et al., 2017).

**Table 4:** Linear mixed-effects models and their respective R syntax

Mixed-effects model	R syntax
1	LmerTest::lmer(DV ~ CS*Trial_num+ (1|ppid))
2	LmerTest::lmer(DV ~ CS*Phase+Trial_num+ (1|ppid))

*Note*: In the column R syntax, DV refers to each dependent variable (see main text for details). *CS* refers to the experimental condition (CS+/CS-). *Trial_num* refers to the trial number across conditions within each phase (linear predictor with one degree of freedom). *Phase* refers to the experimental phase, and *ppid* is the participant variable

## Results

### Experiment 1

**Fig. 3:**
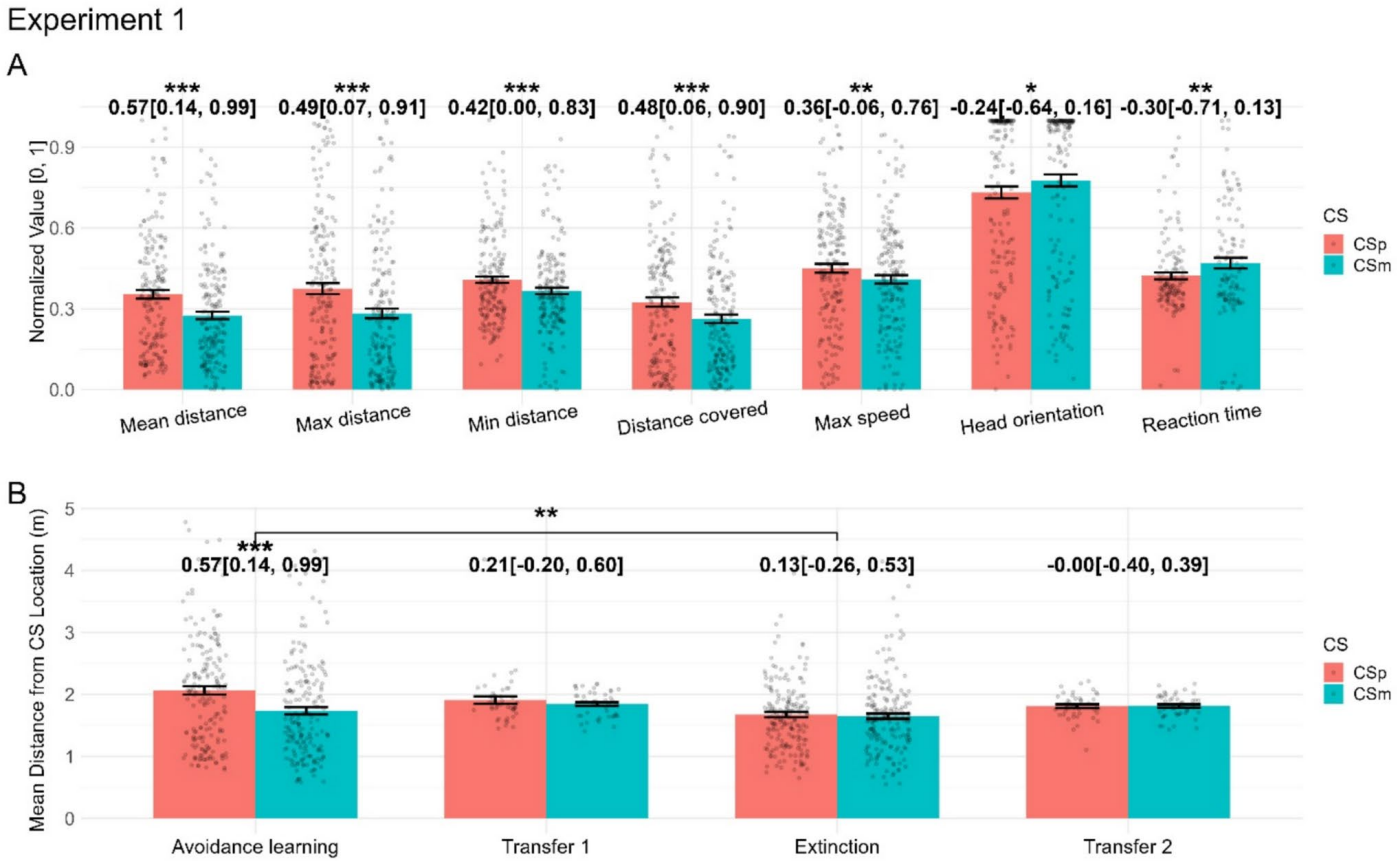
Results from experiment 1. We show all dependent variables for the avoidance learning phase of experiment 1 (**A**) and the main dependent variable for all phases of experiment 1 (**B**). Detailed results can be found in supplementary tables. In panel A, all dependent variables are normalised to the range 0–1 to facilitate illustration. Numbers show Hedge’s *g* with 95% confidence interval for the contrast CS+ vs. CS-, while significance stars reflect *p* values of the condition effect from the within-phase LMM (panel A), and the condition and condition x phase effects from the across-phase LMM (panel B). CSp: CS+, CSm: CS-; *p* < 0.05: *; *p* < 0.01: **; *p* < 0.001: ***

In the avoidance learning phase, all dependent variables differentiated CS+ and CS- with effects sizes (Hedge’s *g*) between 0.2 and 0.6 (Fig. 3A, see supplementary materials on OSF, https://osf.io/yxvfz/, for table of coefficients and *p* values). Of note, the CS+/CS- difference in minimum distance from CS is affected by some participants approaching CS on some trials. The largest effect size was observed for the mean distance from CS. During the first search task, only the maximum distance from CS showed an appreciable condition difference (Fig. 3B). Across the extinction phase, some variables still differentiated CS+ and CS-, but a direct comparison of the avoidance learning and extinction phases indicated extinction (Fig. 3B).

However, effect sizes in the avoidance phase were not as large as expected. Informal debriefing indicated that some participants could not localise the sound and did not find the objectively correct avoidance action. Thus, from experiment 2 onwards, a set of speakers was added to indicate the sound source underneath the pedestal, which vibrated when the US was played.

### Experiment 2

**Fig. 4:**
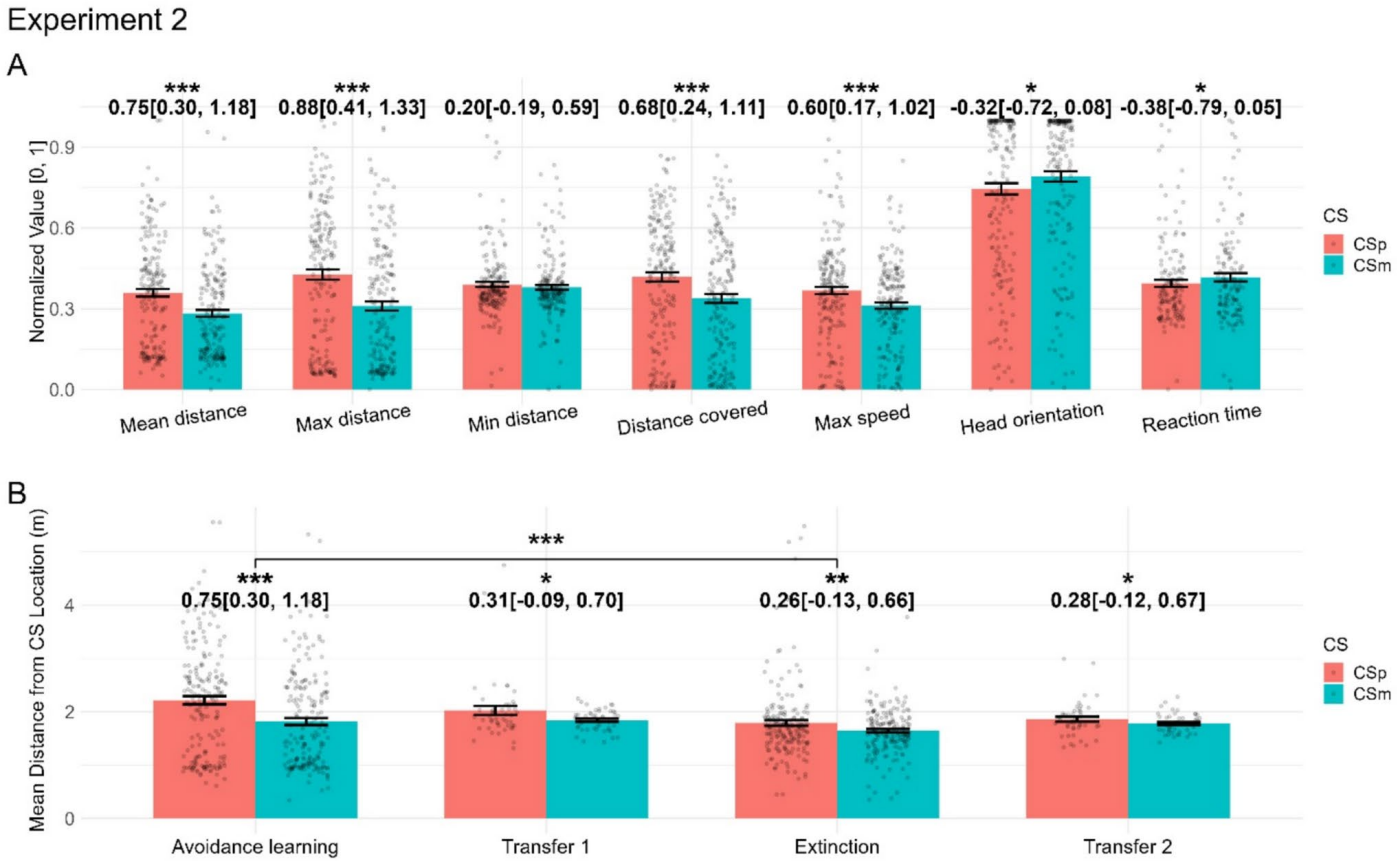
Results from experiment 2. Note that the VR setup was changed between experiments 1 and 2. We show all dependent variables for the avoidance learning phase of experiment 2 (**A**) and the main dependent variable for all phases of experiment 2 (**B**). Detailed results can be found in supplementary tables. In panel A, all dependent variables are normalised to the range 0–1 to facilitate illustration. Numbers show Hedge’s *g* with 95% confidence interval for the contrast CS+ vs. CS-, while significance stars reflect *p* values of the condition effect from the within-phase LMM (panel A), and the condition and condition x phase effects from the across-phase LMM (panel B). *p* < 0.05: *; *p* < 0.01: **; *p* < 0.001: ***

Experiment 2 followed the same design as experiment 1, adding only an indicator of US location. In the avoidance learning phase, all behavioural variables – with the exception of minimum distance from CS – differentiated CS+ and CS-, with generally larger effect sizes than in experiment 1 (Fig. 4A, Table 5 for detailed effect sizes and *F*-statistic). The largest effect was observed for the maximum distance from CS during CS presentation. In the first transfer task, several variables differentiated CS+/CS- (Fig. 4B). Across the extinction phase, some variables still differentiated CS+ and CS-; however, a direct comparison of the avoidance learning and extinction phases indicated extinction.

**Table 5:** Effect sizes with 95% confidence intervals for condition effects, and *F*-statistics for phase interactions, in Experiment 2

Dependent variable	Avoidance learning	Transfer task 1	Extinction	Transfer task 2	Avoidance vs. extinction
Mean distance	*g* = 0.75 [0.30, 1.18] (***)	*g* = 0.31 [– 0.09, 0.70] (*)	*g* = 0.26 [– 0.13, 0.66] (**)	*g* = 0.28 [– 0.12, 0.67] (*)	*F*(1, 740) = 6.19 (***)
Max distance	*g* = 0.88 [0.41, 1.33] (***)	*g* = 0.34 [– 0.06, 0.73](*)	*g* = 0.26 [– 0.14, 0.65] (ns)	*g* = 0.26 [– 0.14, 0.65] (ns)	*F*(1, 740) = 21.74 (***)
Min distance	*g* = 0.20 [– 0.19, 0.59] (ns)	*g* = 0.24 [– 0.15, 0.63] (ns)	*g* = 0.26 [– 0.13, 0.65] (***)	*g* = 0.59 [0.17, 1.01] (**)	*F*(1, 740) = 4.86 (ns)
Distance covered	*g* = 0.68 [0.24, 1.11] (***)	*g* = 0.16 [– 0.23, 0.55] (ns)	*g* = – 0.23 [– 0.62, 0.16] (ns)	*g* = 0.00 [– 0.38, 0.39] (ns)	*F*(1, 740) = 17.27 (***)
Max speed	*g* = 0.60 [0.17, 1.02] (***)	*g* = 0.01 [– 0.38, 0.40] (ns)	*g* = – 0.17 [– 0.56, 0.22] (ns)	*g* = – 0.25 [– 0.64, 0.15] (ns)	*F*(1, 740) = 12.32 (***)
Head direction	*g* = – 0.32 [– 0.72, 0.08] (*)	*g* = 0.42 [0.01, 0.83] (*)	*g* = 0.06 [– 0.33, 0.45] (ns)	*g* = 0.27 [– 0.13, 0.66] (ns)	*F*(1, 740) = 3.71 (*)
Reaction time	*g* = – 0.38 [– 0.79, 0.05] (*)	*g* = – 0.08 [– 0.47, 0.31] (ns)	*g* = 0.24[– 0.15, 0.63] (ns)	*g* = 0.04 [– 0.35, 0.44] (ns)	*F*(1, 635.83) = 3.51 (*)

*Note*: Column headers indicate dependent variables. The first four columns report Hedge’s *g* values with 95% confidence intervals for CS+/CS- difference*,* and the last column report *F*-statistic for phase interaction. Significance stars in the first four columns reflect the *p* values from linear mixed-effects models (LMMs) examining condition effects. Significance stars in the last column reflect *p* values from LMMs examine CS x Phase interaction. *p* < 0.05: *; *p* < 0.01: **; *p* < 0.001: ***; ns = non-significant

Based on the results of experiments 1–2, we chose mean distance from CS as the primary outcome measure for all phases in all following experiments, as it showed large effect sizes and, unlike maximum/minimum distance, is not susceptible to floor/ceiling effects due to the starting point, which is different for different phases. We report all dependent variables in supplementary tables for the sake of completeness.

### Experiment 3

**Fig. 5:**
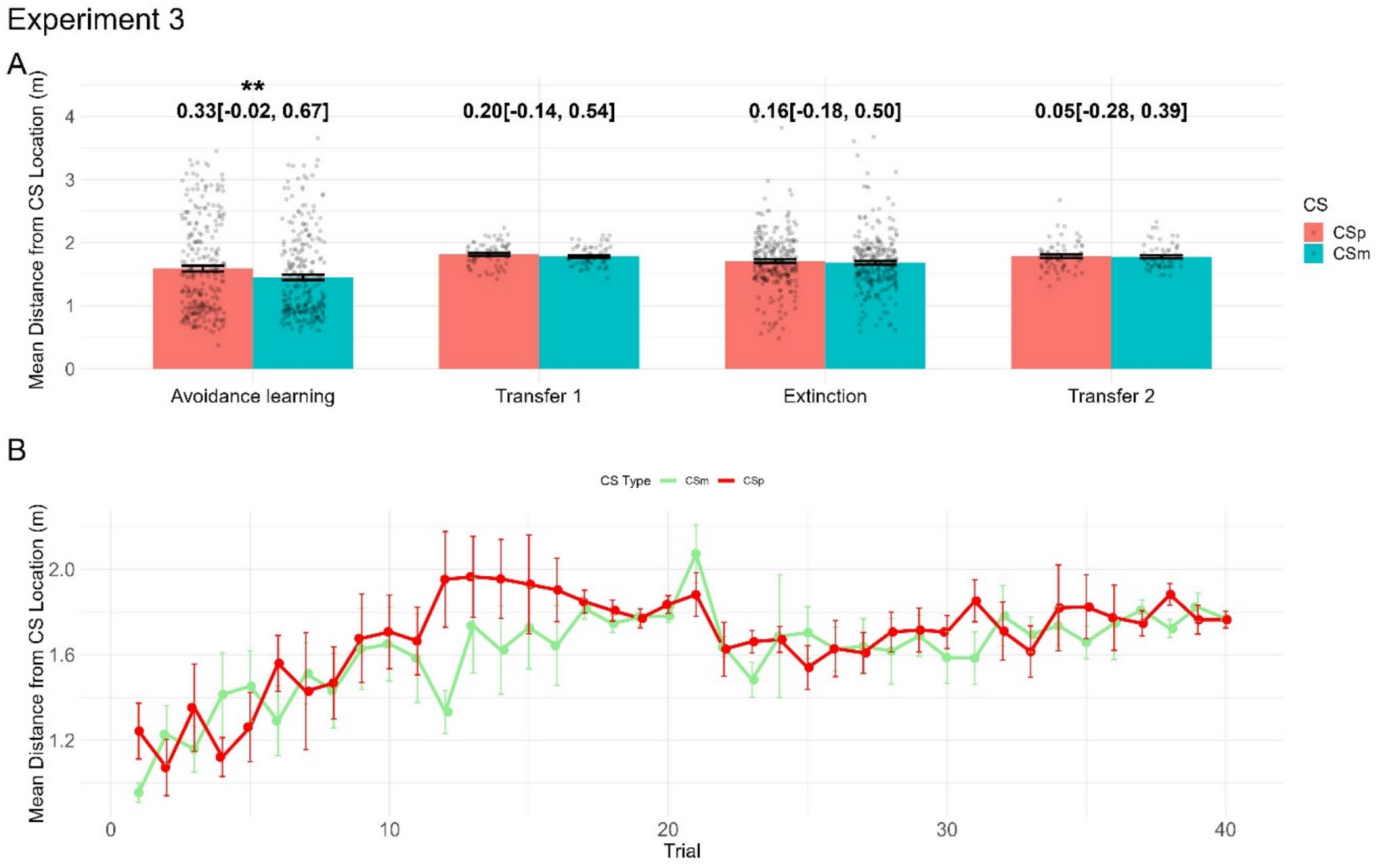
Results from experiment 3. We show the effects of condition (CS+/CS-) for the main dependent variable for all phases of experiment 3 (**A**) and trial-by-trial averages of the main dependent variables for both CS+/CS- conditions for the avoidance learning phase (**B**). Numbers show Hedge’s *g* with 95% confidence interval for the contrast CS+ vs. CS-*,* while significance stars reflect *p* values of the condition effect from the across-phase LMM*.* *p* < 0.05: *; *p* < 0.01: **; *p* < 0.001: ***

With experiment 3, we sought to determine whether avoidance learning takes place without a preceding Pavlovian acquisition phase. In the avoidance learning phase, our primary avoidance measure (mean distance from CS) indicated differentiation of CS+ and CS-, albeit with smaller effect size than in experiment 2 (Fig. 5A). There was no appreciable CS+/CS- difference in any of the variables in the transfer task. A direct comparison of the avoidance learning and extinction phases indicated a reduction of avoidance across CS+/CS- but no CS x phase interaction.

There are two potential reasons for the smaller effect size in the avoidance learning phase: that participants do not avoid as much for the CS+, or that they generalise avoidance to the CS- as well (e.g. perceptual generalisation). Thus, if participants start moving away from all CS in early trials, they might miss the information that no US is delivered on CS- trials. Trial-by-trial data (Fig. 5B) seem to suggest that participants avoid the CS- (even though less than the CS+) for most of the avoidance phase. In line with this, the CS x trial interaction was not significant in the avoidance learning phase, *F*(1, 480.01) = 1.54, *p* = 0.22. To explore the possibility of generalisation, we conducted an additional linear mixed-effects model for CS- with trial number as the independent variable, including random intercepts at the participant level. This exploratory analysis demonstrated a significant main effect of trial number (*F*(1, 224.75) = 65.57, *p* < 0.001), suggesting increased avoidance of the CS- over the acquisition phase. Together, these findings suggest that participants may generalise avoidance learning from CS+ to CS-.

### Experiment 4

**Fig. 6:**
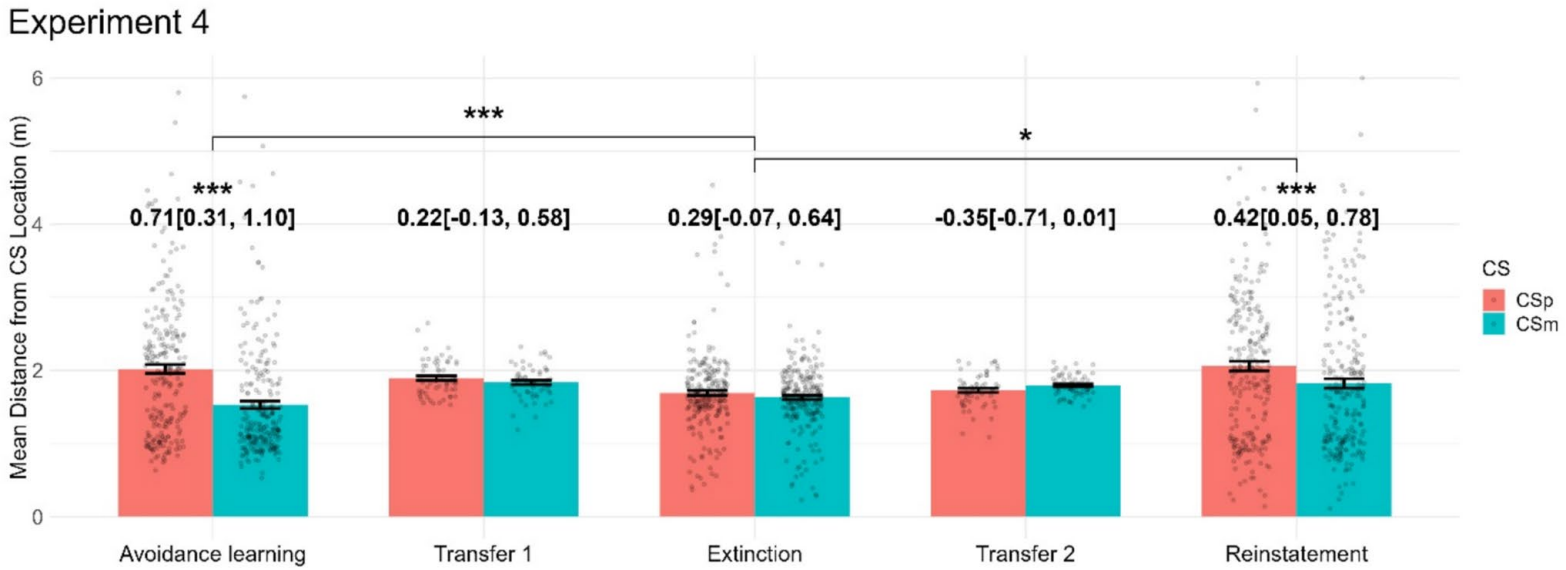
Effects of condition (CS+/CS-) for main dependent variable for all phases in experiment 4. Numbers show Hedge’s *g* with 95% confidence interval for the contrast CS+ vs. CS-*,* while significance stars reflect *p* values of the condition effect from the within-phase LMM, and the condition and condition x phase effects from the across-phase LMM*. p* < 0.05: *; *p* < 0.01: **; *p* < 0.001: ***

Experiment 4 was conducted to confirm out-of-sample generalisation of the results obtained in experiment 2 and followed the same design, with an exploratory reinstatement phase at the end. In the avoidance learning phase, all behavioural variables, including the primary outcome measure indicated differentiation of CS+ and CS- conditions (see supplemental materials for details). There was no CS+/CS- difference in our primary outcome measure in the transfer task or in the extinction phase. A direct comparison of the avoidance learning and extinction phases indicated successful extinction (Fig. 6). After reinstatement, most behavioural variables including the primary outcome measure differentiated CS+/CS-. Direct comparison of the extinction and reinstatement phases indicated successful reinstatement (Fig. 6).

### Experiment 5

**Fig. 7:**
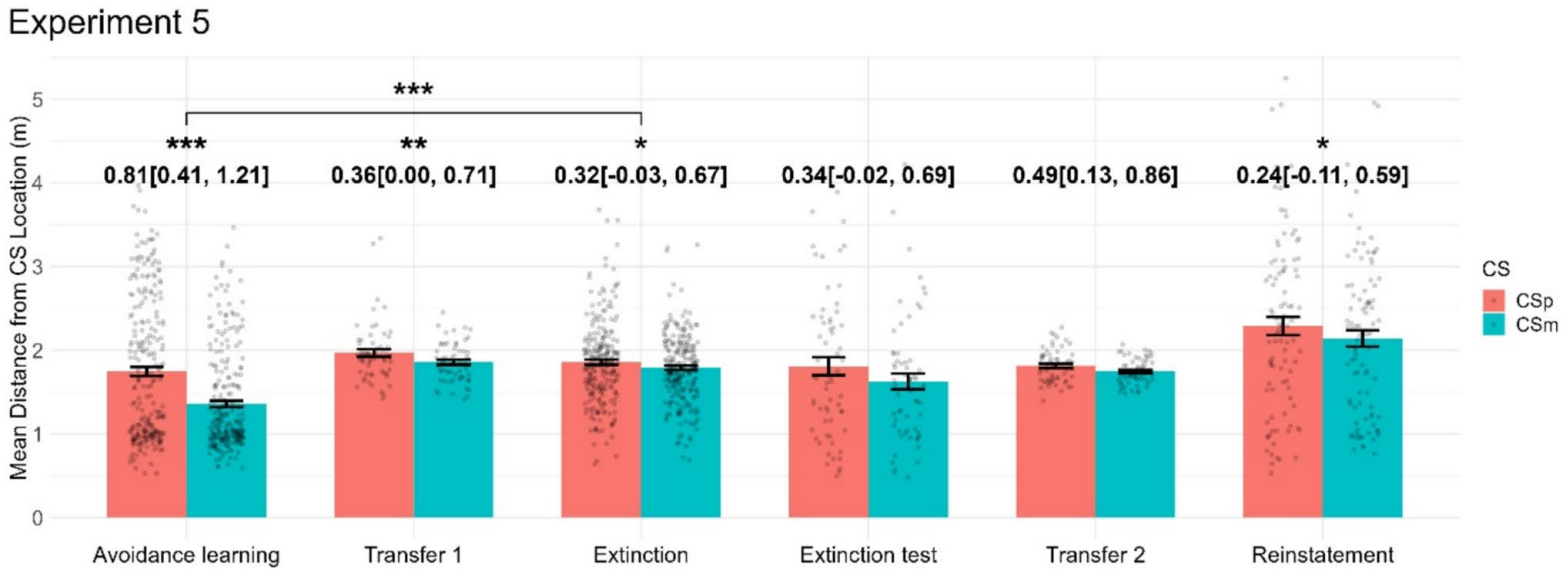
Effects of condition (CS+/CS-) for the main dependent variable across all phases in experiment 5. Numbers show Hedge’s *g* with 95% confidence interval for the contrast CS+ vs. CS-*,* while significance stars reflect *p* values of the condition effect from the within-phase LMM, and the condition and condition x phase effects from the across-phase LMM. *p* < 0.05: *; *p* < 0.01: **; *p* < 0.001: ***

Experiment 5 was conducted to confirm out-of-sample generalisation of the results obtained in experiment 3 and generally followed the same design. As we had suspected generalisation of avoidance to the CS- in experiment 3, we changed the CS to be perceptually more dissimilar (Methods: Stimuli). Furthermore, we added an exploratory extinction recall phase and a reinstatement phase. In the avoidance learning phase, most behavioural measures including the primary outcome measure indicated differentiation of CS+ and CS- condition (see supplementary material). The primary avoidance measure differentiated CS+/CS- the first transfer task, with a larger effect size than in experiment 3. In the extinction phase, the primary avoidance measure still differentiated CS+/CS- but a direct comparison of avoidance learning and extinction phases indicated extinction. In the extinction recall test, there was no differentiation of CS+/CS- in the primary avoidance measure (albeit a small effect was seen in maximum distance). After reinstatement, the primary avoidance measure differentiated CS+/CS- and was numerically higher than during extinction. However, there was no CS x phase interaction, indicating no reinstatement for both the CS+ and CS-. See Fig. 7 for data patterns and see supplementary material for detailed statistical information.

## Discussion

Avoidance learning is a form of instrumental conditioning that enables individuals to evade potentially harmful stimuli and, in its maladaptive form, can be clinically relevant for many mental health disorders. Here, we developed and validated a novel avoidance learning paradigm for humans to study natural and uninstructed avoidance actions, which are assessed in a fine-grained way rather than categorically.

We report three main results. First, across five experiments with slightly different design, participants exhibited the objectively correct avoidance action upon presentation of the CS+. Initial exploratory experiments identified mean distance from CS over the CS-US interval as a sensitive measure that was confirmed in independent samples. Participants avoided the CS+ more than the CS- both when US contingencies were trained in a preceding Pavlovian acquisition phase as well as when they were not. Differential avoidance, however, appeared to be potentially affected by perceptual similarity of CS+ and CS-. Second, action was partly extinguished when CS+ was no longer coupled with US and participants were instructed to approach CS. However, several experiments demonstrated residual avoidance even during this extinction training. Third, one experiment showed latent associations were left intact during extinction, as revealed by return of avoidance after reinstatement. This result, however, was not replicated in a second experiment with different design and an intervening extinction recall phase.

These findings generally validate our paradigm as reproducing several canonical features of avoidance learning (acquisition, extinction) and a classical feature of Pavlovian conditioning (reinstatement). We note that our extinction phase intentionally included a feature of exposure therapy, namely the explicit instruction to approach the CS, as we sought to demonstrate that avoidance learning in our paradigm can be extinguished in principle. Whether extinction would also occur spontaneously remains to be determined and could be informative if one sought to investigate the persistence of maladaptive avoidance. In such a case, it might be useful to revert to the paradigm version used in experiment 1, where no indication on US presence is given once participants walk more than 4 m away from the US. This might be more comparable to naturally occurring situations outside a therapy setting.

Reinstatement after successful extinction was observed in experiment 4 but not experiment 5. Two reasons might account for this. The first is that experiment 4 included a Pavlovian acquisition phase preceding avoidance learning. Reinstatement is classically observed in Pavlovian conditioning and generally thought to reflect the nature of extinction learning as an additional inhibitory association which can neurobiologically tracked to neural structures different from those mediating extinction (Kindt, 2018). Thus, it is possible that reinstatement does not (fully) form for non-Pavlovian avoidance learning. On the other hand, the reinstatement phase in experiment 5 comprised fewer trials than in experiment 4, and trial-by-trial variability could be a methodological reason why reinstatement was not confirmed in experiment 5.

While we succeeded in instating learning, avoidance behaviour was not consistently observed during an incidental transfer task. This task might be improved, for example by including a forced choice task (based on Binder & Spoormaker, 2020).

Results across all experiments confirm that our virtual reality paradigm can indeed be utilised to experimentally induce avoidance behaviour. While this paradigm is presented here in its basic form, it could be further extended and modified to study complex learning phenomena. Despite promising efforts (Maia, 2010; Moutoussis et al., 2008; Palminteri et al., 2012, 2015), cognitive-computational models of avoidance learning are still scarce. Assessing complex learning phenomena (e.g. second-order conditioning, overshadowing, blocking, etc.) relating to avoidance learning would be crucial in developing such models. Therefore, our tool might spawn crucial applications in the future cognitive and computational research of avoidance learning.

To conclude, we present a novel naturalistic VR paradigm that can induce learning and extinction of avoidance behaviours in healthy human participants without instruction or previous knowledge of experimental contingencies. This makes our paradigm a precise, sensitive, largely automatised and highly standardised tool for testing avoidance learning with high ecological validity.

## Supplementary information

Below is the link to the electronic supplementary material.

Supplementary file1 (XLSX 35 KB)

Supplementary file2 (XLSX 50 KB)

## Data Availability

Anonymised data and supplemental materials are publicly available on OSF (https://osf.io/yxvfz/). The CogLearn Toolkit for Unity is publicly available on OSF (https://osf.io/8u9ms/). None of the experiments was preregistered.

## Code availability

R scripts for data-analysis are publicly available on OSF (https://osf.io/yxvfz/).

## References

[CR1] Bach, D. R., Melinščak, F., Fleming, S. M., & Voelkle, M. C. (2020). Calibrating the experimental measurement of psychological attributes. *Nature Human Behaviour,**4*(12), 1229–1235. 10.1038/s41562-020-00976-8 10.1038/s41562-020-00976-8 33199857

[CR2] Binder, F. P., & Spoormaker, V. I. (2020). Quantifying Human Avoidance Behavior in Immersive Virtual Reality. *Frontiers in Behavioral Neuroscience*, *14*. 10.3389/fnbeh.2020.569899 10.3389/fnbeh.2020.569899 PMC7554565 33192365

[CR3] Brookes, J., Warburton, M., Alghadier, M., Mon-Williams, M., & Mushtaq, F. (2020). Studying human behavior with virtual reality: The Unity Experiment Framework. *Behavior Research Methods,**52*(2), 455–463. 10.3758/s13428-019-01242-0 31012061 10.3758/s13428-019-01242-0 PMC7148262

[CR4] Brookes, J., Hall, S., Frühholz, S., & Bach, D. R. (2023). Immersive VR for investigating threat avoidance: The VRthreat toolkit for Unity. *Behavior Research Methods*. 10.3758/s13428-023-02241-y 10.3758/s13428-023-02241-y PMC11289213 37794208

[CR5] Colwill, R. M. (2022). Avoidance. In J. Vonk & T. K. Shackelford (Eds.), *Encyclopedia of Animal Cognition and Behavior* (pp. 590–594). Springer International Publishing. 10.1007/978-3-319-55065-7_1033

[CR6] Craske, M. G., Stein, M. B., Eley, T. C., Milad, M. R., Holmes, A., Rapee, R. M., & Wittchen, H.-U. (2017). Anxiety disorders. *Nature Reviews Disease Primers,**3*(1), 1–19. 10.1038/nrdp.2017.24 10.1038/nrdp.2017.24 PMC11009418 28470168

[CR7] De Wit, S., Kindt, M., Knot, S. L., Verhoeven, A. A. C., Robbins, T. W., Gasull-Camos, J., Evans, M., Mirza, H., & Gillan, C. M. (2018). Shifting the balance between goals and habits: Five failures in experimental habit induction. *Journal of Experimental Psychology: General,**147*(7), 1043–1065. 10.1037/xge0000402 29975092 10.1037/xge0000402 PMC6033090

[CR8] Faul, F., Erdfelder, E., Lang, A.-G., & Buchner, A. (2007). G*Power 3: A flexible statistical power analysis program for the social, behavioral, and biomedical sciences. *Behavior Research Methods,**39*(2), 175–191. 10.3758/BF03193146 17695343 10.3758/bf03193146

[CR9] Fergus, T. A., & Valentiner, D. P. (2009). The disgust propensity and sensitivity scale–revised: An examination of a reduced-item version. *Journal of Anxiety Disorders,**23*(5), 703–710. 10.1016/j.janxdis.2009.02.009 19278821 10.1016/j.janxdis.2009.02.009

[CR10] Flores, A., López, F. J., Vervliet, B., & Cobos, P. L. (2018). Intolerance of uncertainty as a vulnerability factor for excessive and inflexible avoidance behavior. *Behaviour Research and Therapy,**104*, 34–43. 10.1016/j.brat.2018.02.008 29524740 10.1016/j.brat.2018.02.008

[CR11] Glogan, E., Meulders, M., Pfeiffer, L., Vlaeyen, J. W. S., & Meulders, A. (2022). Alike, but not quite: Comparing the generalization of pain-related fear and pain-related avoidance. *The Journal of Pain,**23*(9), 1616–1628. 10.1016/j.jpain.2022.04.010 35508274 10.1016/j.jpain.2022.04.010

[CR12] Godier, L. R., de Wit, S., Pinto, A., Steinglass, J. E., Greene, A. L., Scaife, J., Gillan, C. M., Walsh, B. T., Simpson, H.-B., & Park, R. J. (2016). An investigation of habit learning in anorexia nervosa. *Psychiatry Research,**244*, 214–222. 10.1016/j.psychres.2016.07.051 27497292 10.1016/j.psychres.2016.07.051 PMC5718042

[CR13] Grös, D. F., Antony, M. M., Simms, L. J., & McCabe, R. E. (2007). Psychometric properties of the State-Trait Inventory for Cognitive and Somatic Anxiety (STICSA): Comparison to the State-Trait Anxiety Inventory (STAI). *Psychological Assessment,**19*(4), 369–381. 10.1037/1040-3590.19.4.369 18085930 10.1037/1040-3590.19.4.369

[CR14] Harris, P. A., Taylor, R., Thielke, R., Payne, J., Gonzalez, N., & Conde, J. G. (2009). Research electronic data capture (REDCap)—A metadata-driven methodology and workflow process for providing translational research informatics support. *Journal of Biomedical Informatics,**42*(2), 377–381. 10.1016/j.jbi.2008.08.010 18929686 10.1016/j.jbi.2008.08.010 PMC2700030

[CR15] Hoyle, R. H., Stephenson, M. T., Palmgreen, P., Lorch, E. P., & Donohew, R. L. (2002). Reliability and validity of a brief measure of sensation seeking. *Personality and Individual Differences,**32*(3), 401–414. 10.1016/S0191-8869(01)00032-0

[CR16] Kennedy, R. S., Lane, N. E., Berbaum, K. S., & Lilienthal, M. G. (1993). Simulator Sickness Questionnaire: An enhanced method for quantifying simulator sickness. *The International Journal of Aviation Psychology,**3*(3), 203–220. 10.1207/s15327108ijap0303_3

[CR17] Kindt, M. (2018). The surprising subtleties of changing fear memory: A challenge for translational science. *Philosophical Transactions of the Royal Society B: Biological Sciences,**373*(1742), 20170033. 10.1098/rstb.2017.0033 10.1098/rstb.2017.0033 PMC5790831 29352032

[CR18] Krypotos, A.-M. (2015). Avoidance learning: A review of theoretical models and recent developments. *Frontiers in Behavioral Neuroscience*, *9*. 10.3389/fnbeh.2015.00189 10.3389/fnbeh.2015.00189 PMC4508580 26257618

[CR19] Krypotos, A.-M., Vervliet, B., & Engelhard, I. M. (2018). The validity of human avoidance paradigms. *Behaviour Research and Therapy,**111*, 99–105. 10.1016/j.brat.2018.10.011 30396111 10.1016/j.brat.2018.10.011

[CR20] Kuznetsova, A., Brockhoff, P. B., & Christensen, R. H. B. (2017). lmerTest Package: Tests in linear mixed effects models. *Journal of Statistical Software,**82*, 1–26. 10.18637/jss.v082.i13

[CR21] Lemmens, A., Beckers, T., Dibbets, P., Kang, S., & Smeets, T. (2021). Overgeneralization of fear, but not avoidance, following acute stress. *Biological Psychology,**164*, 108151. 10.1016/j.biopsycho.2021.108151 34302889 10.1016/j.biopsycho.2021.108151

[CR22] Maia, T. V. (2010). Two-factor theory, the actor-critic model, and conditioned avoidance. *Learning & Behavior,**38*(1), 50–67. 10.3758/LB.38.1.50 20065349 10.3758/LB.38.1.50

[CR23] Moutoussis, M., Bentall, R. P., Williams, J., & Dayan, P. (2008). A temporal difference account of avoidance learning. *Network: Computation in Neural Systems,**19*(2), 137–160. 10.1080/09548980802192784 18569725 10.1080/09548980802192784

[CR24] Norbury, A., Robbins, T. W., & Seymour, B. (2018). Value generalization in human avoidance learning. *eLife,**7*, e34779. 10.7554/eLife.34779 29735014 10.7554/eLife.34779 PMC5957527

[CR25] Palminteri, S., Justo, D., Jauffret, C., Pavlicek, B., Dauta, A., Delmaire, C., Czernecki, V., Karachi, C., Capelle, L., Durr, A., & Pessiglione, M. (2012). Critical roles for anterior insula and dorsal striatum in punishment-based avoidance learning. *Neuron,**76*(5), 998–1009. 10.1016/j.neuron.2012.10.017 23217747 10.1016/j.neuron.2012.10.017

[CR26] Palminteri, S., Khamassi, M., Joffily, M., & Coricelli, G. (2015). Contextual modulation of value signals in reward and punishment learning. *Nature Communications,**6*(1), 8096. 10.1038/ncomms9096 26302782 10.1038/ncomms9096 PMC4560823

[CR27] Patterson, T. K., Craske, M. G., & Knowlton, B. J. (2019). Enhanced avoidance habits in relation to history of early-life stress. *Frontiers in Psychology*, *10*. 10.3389/fpsyg.2019.01876 10.3389/fpsyg.2019.01876 PMC6700232 31456726

[CR28] Pittig, A., Treanor, M., LeBeau, R. T., & Craske, M. G. (2018). The role of associative fear and avoidance learning in anxiety disorders: Gaps and directions for future research. *Neuroscience & Biobehavioral Reviews,**88*, 117–140. 10.1016/j.neubiorev.2018.03.015 29550209 10.1016/j.neubiorev.2018.03.015

[CR29] R Core Team. (2022). *R: A Langua ge and Environment for Statistical Computing*. R Foundation for Statistical Computing. https://www.R-project.org/

[CR30] Ree, M. J., French, D., MacLeod, C., & Locke, V. (2008). Distinguishing cognitive and somatic dimensions of state and trait anxiety: Development and validation of the State-Trait Inventory for Cognitive and Somatic Anxiety (STICSA). *Behavioural and Cognitive Psychotherapy,**36*(3), 313–332. 10.1017/S1352465808004232

[CR31] Reichenberger, J., Porsch, S., Wittmann, J., Zimmermann, V., & Shiban, Y. (2017). Social fear conditioning paradigm in virtual reality: Social vs. electrical aversive conditioning. *Frontiers in Psychology*, *8*. 10.3389/fpsyg.2017.01979 10.3389/fpsyg.2017.01979 PMC5715328 29250000

[CR32] Roberts, C., Apergis-Schoute, A. M., Bruhl, A., Nowak, M., Baldwin, D. S., Sahakian, B. J., & Robbins, T. W. (2022). Threat reversal learning and avoidance habits in generalised anxiety disorder. *Translational Psychiatry,**12*(1), 1–7. 10.1038/s41398-022-01981-3 35641488 10.1038/s41398-022-01981-3 PMC9156703

[CR33] San Martín, C., Jacobs, B., & Vervliet, B. (2020). Further characterization of relief dynamics in the conditioning and generalization of avoidance: Effects of distress tolerance and intolerance of uncertainty. *Behaviour Research and Therapy,**124*, 103526. 10.1016/j.brat.2019.103526 31778930 10.1016/j.brat.2019.103526

[CR34] Sporrer, J. K., Brookes, J., Hall, S., Zabbah, S., Serratos Hernandez, U. D., & Bach, D. R. (2023). Functional sophistication in human escape. *iScience,**26*(11), 108240. 10.1016/j.isci.2023.108240 38026199 10.1016/j.isci.2023.108240 PMC10654542

[CR35] Stein, D. J., Costa, D. L. C., Lochner, C., Miguel, E. C., Reddy, Y. C. J., Shavitt, R. G., van den Heuvel, O. A., & Simpson, H. B. (2019). Obsessive–compulsive disorder. *Nature Reviews Disease Primers,**5*(1), 1–21. 10.1038/s41572-019-0102-3 10.1038/s41572-019-0102-3 PMC7370844 31371720

[CR36] Tolchinsky, A. (2013). The development of a self-report questionnaire to measure problematic video game play and its relationship to other psychological phenomena. *Master’s Theses and Doctoral Dissertations*. https://commons.emich.edu/theses/555

[CR37] Wolpe, J., & Lang, P. J. (1964). A fear survey schedule for use in behaviour therapy. *Behaviour Research and Therapy,**2*(1), 27–30. 10.1016/0005-7967(64)90051-8 14170305 10.1016/0005-7967(64)90051-8

[CR38] Wong, A. H. K., & Pittig, A. (2022). Avoiding a feared stimulus: Modelling costly avoidance of learnt fear in a sensory preconditioning paradigm. *Biological Psychology,**168*, 108249. 10.1016/j.biopsycho.2021.108249 34973369 10.1016/j.biopsycho.2021.108249

[CR39] Yehuda, R., Hoge, C. W., McFarlane, A. C., Vermetten, E., Lanius, R. A., Nievergelt, C. M., Hobfoll, S. E., Koenen, K. C., Neylan, T. C., & Hyman, S. E. (2015). Post-traumatic stress disorder. *Nature Reviews Disease Primers,**1*, 15057. 10.1038/nrdp.2015.57 27189040 10.1038/nrdp.2015.57

